# Inflammatory myofibroblastic tumor of the pleura with adjacent chest wall invasion and metastasis to the kidney: a case report

**DOI:** 10.1186/s13256-018-1796-7

**Published:** 2018-09-09

**Authors:** Yong-sub Na, Sang-gon Park

**Affiliations:** 1 Department of Pulmonology, Chosun University Hospital, Gwangju, Republic of Korea; 2 Department of Hemato-oncology, Chosun University Hospital, 365 Pilmun-daero, Dong-gu, Gwangju, 61453 Republic of Korea

**Keywords:** Inflammatory myofibroblastic tumor, Pleura, Metastasis, Kidney, Treatment

## Abstract

**Background:**

Inflammatory myofibroblastic tumor is a rare benign neoplasm that frequently involves the lung and abdominopelvic region, and is found mainly in children and young adults. Inflammatory myofibroblastic tumor tends to be locally invasive or recurrent, and rarely metastasizes.

**Case presentation:**

A 76-year-old Korean man presented with a history of upper back pain for 2 months and motor weakness in both lower extremities for 2 days. Contrast-enhanced computed tomography of his chest and abdomen showed a large heterogeneous pleural mass involving the right fifth rib and vertebral body and a mass infiltrating the right renal hilum. Computed tomography-guided percutaneous needle biopsy of the pleural mass was performed. The histological findings on hematoxylin and eosin staining showed proliferation of spindle cells with infiltration of lymphocytes and plasma cells. Immunohistochemistry showed neoplastic cells positive for CD68, focally positive for smooth muscle actin, and negative for cytokeratin and desmin. Inflammatory myofibroblastic tumor was diagnosed based on the histological examination. Treatment with glucocorticoids (methylprednisolone 1 mg/kg) and radiotherapy (5 days/week for 3 weeks at 3 Gy/fraction, 45 Gy/15 days) was started. After 1 month, chest computed tomography showed a reduction in the size of the pleural mass, and abdominopelvic computed tomography showed decreased infiltration around the right renal pelvis.

**Conclusions:**

Inflammatory myofibroblastic tumor is a rare neoplasm of intermediate malignant potential due to a tendency for local recurrence and it rarely develops distant metastases. Complete surgical resection is the primary treatment. However, unresectable and metastatic inflammatory myofibroblastic tumor can be treated with systemic therapy, including glucocorticoids, radiotherapy, and/or chemotherapy.

## Background

Inflammatory myofibroblastic tumor (IMT) is a rare benign neoplasm that frequently involves the lung and abdominopelvic region, and is mainly found in children and young adults [1]. The histological features of IMT include proliferation of spindle cells with a prominent inflammatory infiltrate consisting of plasma cells and lymphocytes, with occasional eosinophils and neutrophils [2]. IMT tends to be locally invasive or recurrent, and rarely metastasizes [3]. Pulmonary IMT most commonly presents as a solitary pulmonary nodule or well-circumscribed mass on chest radiography [4, 5], but rarely presents in the pleura [6].

Here we present the case of a 76-year-old Korean man with pleural IMT involving the adjacent chest wall, with metastasis to the kidney.

## Case presentation

A 76-year-old Korean man presented with upper back pain for 2 months and motor weakness in both lower extremities for 2 days. A chest radiograph and computed tomography (CT) performed at another institution showed a pleural mass in the upper lobe of his right lung. He was referred to our hospital for evaluation of the pleural mass and paraplegia. He had been treated with medication for 4 years for type 2 diabetes mellitus and primary hypertension. He did not have a family history of malignant disease. On chest examination, he had tenderness at the level of the fifth rib on the right side. On neurologic examination, he showed paraplegia with numbness and a sensory deficit below the T5 dermatome.

Laboratory findings showed an elevated C-reactive protein level of 6.84 mg/dL (normal range, 0.0–0.3 mg/dL), blood urea nitrogen of 25 mg/dL (normal range, 7.8–22.0 mg/dL), and a serum creatinine of 1.5 mg/dL (normal range, 0.6–1.4 mg/dL). Urine analysis showed 4+ blood, with many red blood cells and 0–1 white blood cells/high-power field (Table 1).

**Table 1:** Laboratory data at admission and follow-up

Variable	Admission	HD #18	HD #28
WBCs (× 10^3^/μL)	10.17	7.22	4.99
Hb (g/dL)	14.1	11.8	12.3
Platelets (×10^3^/μL)	243	219	126
ESR (mm/hour)	29	13	14
CRP (mg/dL)	6.84	0.55	0.64
BUN (mg/dL)	25	22.5	15.0
Creatinine (mg/dL)	1.5	0.81	0.82
Urinalysis			
Blood	4+	Negative	Negative
Protein	+/−	Negative	Negative
Urine microscopy			
Red blood cells	Many/hpf	1–4/hpf	1–4/hpf
White blood cells	0–1/hpf	10–19/hpf	5–9/hpf
Clinical course	Hydration Biopsy	Steroid therapy Radiotherapy	Steroid therapy

*BUN* blood urea nitrogen, *CRP* C-reactive protein, *ESR* erythrocyte sedimentation rate, *Hb* hemoglobin, *HD* hospital day, *hpf* high-power field, *WBC* white blood cell

**Fig. 1:**
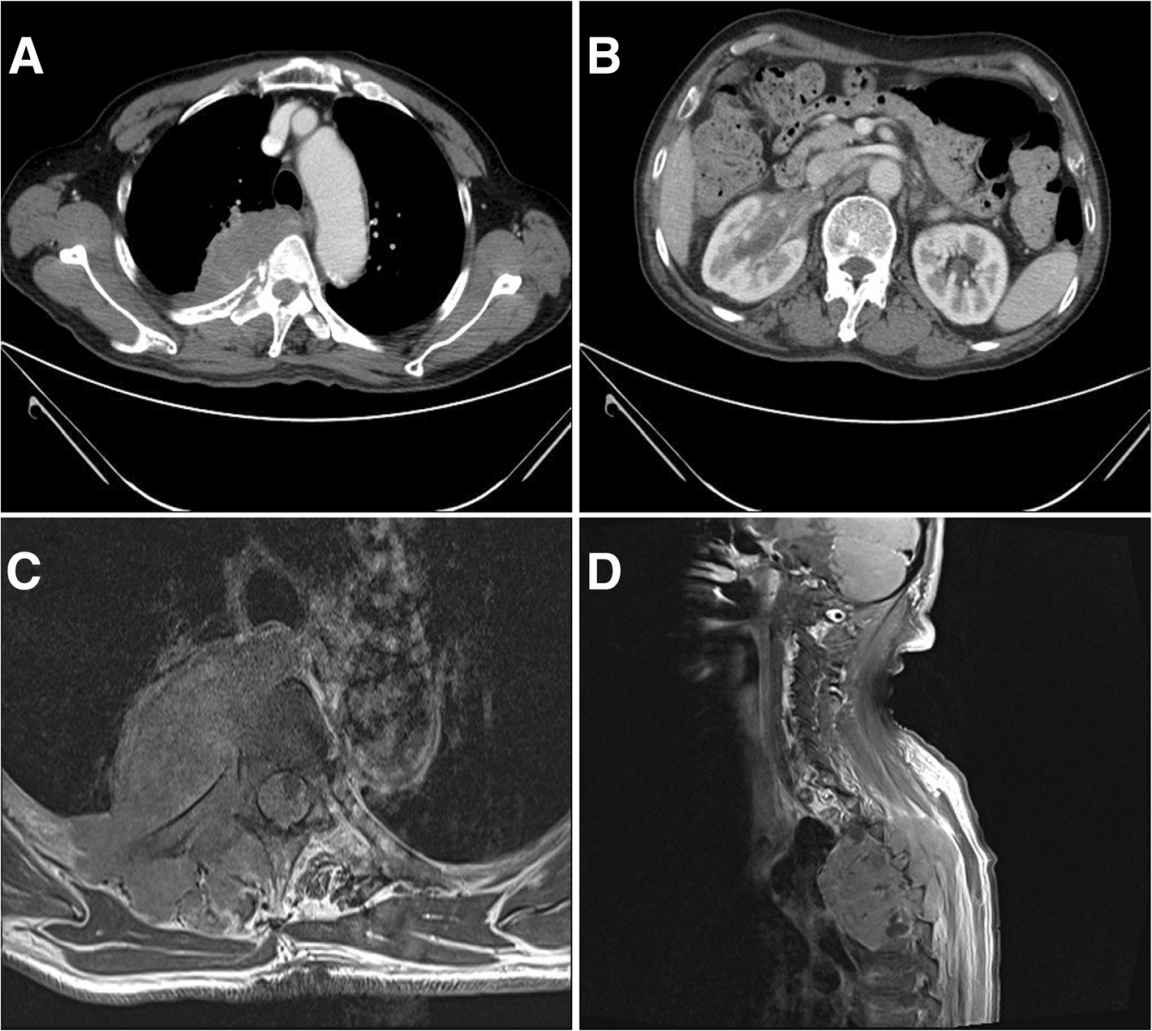
Contrast-enhanced computed tomography and magnetic resonance imaging study at admission. **a** Contrast-enhanced chest computed tomography shows a large heterogeneous pleural mass involving the right fifth rib and vertebral body. **b** Contrast-enhanced abdominopelvic computed tomography shows a mass infiltrating the right renal hilum (longest diameter, 44 mm). **c** Axial T1-weighted magnetic resonance imaging shows a pleural mass with high signal intensity extending to rib, muscle, and vertebral body. **d** Sagittal T1-weighted magnetic resonance imaging shows a pleural mass with high signal intensity in the right paravertebral area at the level of T3 to T6

A chest radiograph showed a large homogenous opacity in the right superior mediastinum. Contrast-enhanced chest CT showed a 7.3 cm × 4.4 cm × 7.7 cm heterogeneous pleural mass involving the right fifth rib and vertebral body (Fig. 1a).

Contrast-enhanced abdominopelvic CT showed a mass infiltrating the right renal hilum without vascular occlusion or hydronephrosis (Fig. 1b). Spine CT and enhanced magnetic resonance imaging (MRI) showed a large pleural mass in the right paravertebral area at the level of T3 to T6 (Fig. 1c, d). CT-guided percutaneous needle biopsy of the pleural mass was performed.

**Fig. 2:**
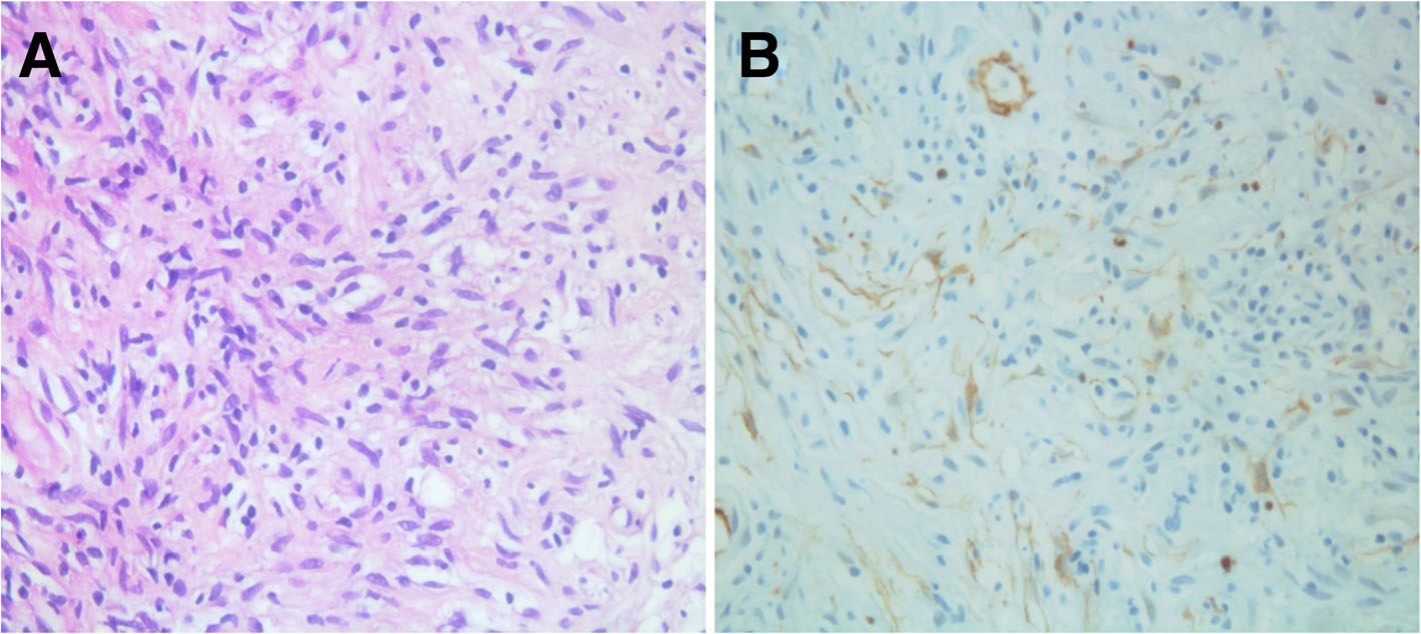
**a** Histological examination (hematoxylin-eosin staining) shows spindle cell proliferation with infiltration of lymphocytes and plasma cells. **b** Immunohistochemically, neoplastic cells are focally positive for smooth muscle actin

Histological findings on hematoxylin and eosin (H&E) staining showed proliferation of spindle cells with infiltration of lymphocytes and plasma cells (Fig. 2a). Immunohistochemistry showed neoplastic cells positive for CD68, focally positive for smooth muscle actin (SMA), and negative for cytokeratin and desmin (Fig. 2b). IMT was diagnosed based on the histological examination.

**Fig. 3:**
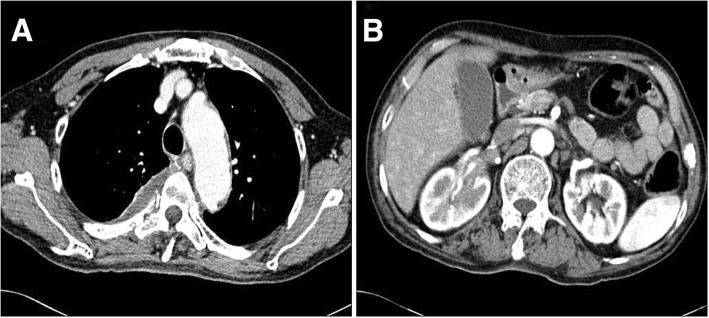
Contrast-enhanced computed tomography after 1 month of treatment. **a** Contrast-enhanced chest computed tomography shows reduction in size of the pleural mass. **b** Contrast-enhanced abdominopelvic computed tomography shows decreased infiltration around the right renal pelvis (longest diameter, 31 mm)

As the tumor could not be completely resected, treatment with glucocorticoids (methylprednisolone 1 mg/kg) and radiotherapy (5 days/week for 3 weeks at 3 Gy/fraction, 45 Gy/15 days) was started. After 1 month, laboratory findings were unremarkable. Hematuria, which is thought to be caused by kidney metastasis of IMT improved after treatment (Table 1). Chest CT showed reduction in the size of the pleural mass (6.0 cm × 1.8 cm × 5.3 cm) and abdominopelvic CT showed decreased infiltration around the right renal pelvis (Fig. 3). The summed-up diameters of both target lesions decreased from 121 mm to 91 mm, that is, a 24.8% reduction of baseline diameter. According to Response Evaluation Criteria in Solid Tumors (RECIST) 1.1 criteria it could be defined as stable disease. He was discharged on orally administered glucocorticoids and showed improvement in symptoms on follow-up 1 month after hospital discharge. Since then, however, he has not attended a follow-up visit.

## Discussion

IMT is a mesenchymal neoplasm composed of myofibroblastic and fibroblastic spindle cells accompanied with an inflammatory infiltrate of plasma cells, lymphocytes, and/or eosinophils; it mainly affects children and young adults. IMT has also been known as fibrous histiocytoma, inflammatory pseudotumor, xanthogranuloma, fibroxanthoma, plasma cell granuloma, and inflammatory fibrosarcoma [3]. IMT most commonly occurs in the lung, but can also appear in the mesentery, omentum, retroperitoneum, pelvis, abdominal soft tissue, mediastinum, head and neck, upper respiratory tract, trunk, and extremities [1]. However, pleural involvement is rare and has only been described in a few case reports [6–8]. In our case, pleural IMT invaded the adjacent chest wall; vertebral body invasion and metastasis to the kidney were also present.

The etiology and pathogenesis are unknown. Several theories have suggested involvement of the immunologic response to infectious agents such as Epstein–Barr virus or human herpesvirus 8, or an inflammatory reaction to an underlying low-grade malignancy [9]. Griffin *et al.* reported three cases of IMT with chromosomal rearrangements involving the anaplastic lymphoma kinase (ALK) locus at 2p23 [10]. These findings suggest that IMT is a neoplastic rather than reactive subset [10]. ALK-positive IMTs may be associated with local recurrence and favorable prognosis, but not metastasis [11].

In our case, the patient presented upper back pain and motor weakness in both lower extremities due to IMT involving pleura and vertebra body. Laboratory findings showed an elevated erythrocyte sedimentation rate and elevated C-reactive protein. In addition, urine analysis showed hematuria at admission due to metastasis to the kidney. The clinical features are determined using the location of IMT, with 15% to 30% of patients presenting with fever, weight loss, and malaise. Laboratory findings include an elevated erythrocyte sedimentation rate, elevated C-reactive protein, microcytic hypochromic anemia, and thrombocytosis [12]. Signs and symptoms of kidney IMT may include flank pain, hydronephrosis, and hematuria [13].

Imaging findings vary according to the site of involvement. Pulmonary IMTs usually present with a solitary, peripheral, circumscribed, lobulated mass on chest radiography. Chest CT shows heterogeneous attenuation and enhancement. An amorphous, mixed, or fine fleck-like pattern of calcification has been reported in pulmonary IMTs. MRI shows homogeneous or heterogeneous signal intensity on T1-weighted and T2-weighted images. Kidney IMTs show homogeneous or cystic masses on enhanced CT [13–15]. Enhanced CT of pleural IMT has been described as showing hypodense or heterogeneous masses [6–8]. Our case showed a heterogeneous pleural mass in the contrast-enhanced chest CT and a pleural mass with high signal intensity in T1-weighted MRI. Fluorodeoxyglucose-positron emission tomography (FDG-PET)/CT of IMT showed variable FDG uptake according to tumor cellularity, tumor cell biological behavior, and the extent of activation of inflammatory cells [16].

The most accurate diagnostic method is surgical excision of the lesion. Fine-needle aspiration is often unable to diagnose the disease because specimens are aseptic and show a mixture of inflammatory cells [5]. The diagnosis of IMT requires identification of characteristic pathological features. The histological features of IMT include variable spindle cell proliferation in a myxoid-to-collagenous stroma with a prominent inflammatory infiltrate composed primarily of plasma cells and lymphocytes, with occasional admixed eosinophils and neutrophils. Immunohistochemical investigation is not required to confirm the diagnosis because of the variable expression and lack of specificity of myofibroblastic markers [2].

The treatment of choice for IMT is complete surgical resection. In this case report, our patient was unable to undergo complete surgical resection because IMT invaded the adjacent chest wall. So, if patients have contraindications to surgical resection, or have unresectable or locally aggressive disease, glucocorticoids, chemotherapy, and radiotherapy can be used. Steroid therapy has shown good response in many studies. Various cytotoxic drugs or regimens (vincristine, cyclophosphamide, doxorubicin, 5-fluorouracil, cisplatin, carboplatin, paclitaxel, methotrexate, vinorelbine, ifosfamide, and etoposide) have been used for treatment with unresectable and/or relapsed IMT in a case series. Recently, Maruyama *et al.* suggested that vinorelbine and methotrexate is a therapeutic option for adult patients with relapsed and unresectable IMT [17]. Radiotherapy may be considered for patients with postoperative recurrences and unresectable IMT [18]. However, there was a lack of clinical evidence and efficacy; also, failures of these modalities have been reported [5, 19, 20]. Although we could not do a long-term follow-up, our case observed a decrease in the size of the mass with radiotherapy and corticosteroid therapy. Butrynski *et al.* reported a sustained partial response to the ALK inhibitor crizotinib in a patient with ALK-translocated IMT [21].

The recurrence rate varies according to the site of involvement, from < 2% for tumors confined to the lung to 25% for extrapulmonary IMTs associated with an abdominopelvic site, multinodular tumor masses, and incomplete resection. Distant metastasis is rare [2, 3]. Development of metastasis may be more frequent in the absence of ALK reactivity [11].

## Conclusions

We presented a rare case of pleural IMT involving the adjacent chest wall and kidney metastasis. IMT is a rare neoplasm of intermediate malignant potential, with a tendency toward local recurrence and rare distant metastasis. Complete surgical resection of IMT is the primary treatment. However, unresectable and metastatic IMT can be treated with systemic therapy, including glucocorticoids, radiotherapy, and/or chemotherapy.

## Abbreviations

ALK: Anaplastic lymphoma kinase
CT: Computed tomography
FDG-PET: Fluorodeoxyglucose-positron emission tomography
H&E: Hematoxylin and eosin
IMT: Inflammatory myofibroblastic tumor
MRI: Magnetic resonance imaging
RECIST: Response Evaluation Criteria in Solid Tumors
SMA: Smooth muscle actin
